# The impact of psychiatric decision units on mental health crisis care pathways: a synthetic control study

**DOI:** 10.1371/journal.pmen.0000171

**Published:** 2025-05-02

**Authors:** Paris Pariza, Izzy Hatfield, Lucy P. Goldsmith, Xiaochen Ge, Jared G. Smith, Katie Anderson, Chloe Crowe, Heather Jarman, Sonia Johnson, Jo Lomani, David McDaid, A.-La Park, Kati J. Turner, Geraldine M. Clarke, Steve Gillard

**Affiliations:** 1 NHS England and NHS Improvement, London, United Kingdom; 2 Improvement Analytics Unit, The Health Foundation, London, United Kingdom; 3 School of Health and Medical Sciences, City St George’s, University of London, London, United Kingdom; 4 Population Health Research Institute, City St George’s, University of London, London, United Kingdom; 5 Clinical Research Unit, South West London and St George’s Mental Health Trust, London, United Kingdom; 6 Department of Psychology, Middlesex University, London, United Kingdom; 7 North East London NHS Foundation Trust, London, United Kingdom; 8 St George’s University Hospitals NHS Foundation Trust, London, United Kingdom; 9 Division of Psychiatry, University College London, London, United Kingdom; 10 Care Policy and Evaluation Centre, London School of Economics and Political Science, London, United Kingdom; PLOS: Public Library of Science, UNITED KINGDOM OF GREAT BRITAIN AND NORTHERN IRELAND

## Abstract

Psychiatric crisis care is under great pressure, with the number of psychiatric presentations to emergency departments increasing and inpatient wards operating with occupancy rates above recommended levels. Internationally, hospital-based short-stay crisis units (named Psychiatric Decision Units; (PDU) in the UK) have been introduced to address these challenges, but the current evidence for their effectiveness is limited. We estimated the effects of PDUs in four geographic locations in England, linked to three National Health Service (NHS) mental health trusts and six NHS acute hospital trusts. Using national data sets to create synthetic controls from areas without PDUs (following the generalised synthetic control method), we estimated trust-wide changes to the primary outcomes of psychiatric inpatient admissions and psychiatric presentations to emergency departments (ED), compared to the synthetic controls, alongside secondary outcomes. We used meta-analysis to robustly combine outcomes. We analysed NHS hospital activity data for adults aged between 18 and 75 years covering 24 months preceding and following the introduction of each PDU (November 2012 to January 2021). We found no significant impacts of PDUs on primary outcomes, except at Sheffield Teaching Hospitals NHS Foundation Trust with 1.5 fewer psychiatric presentations to ED per 10,000 trust population per month (relative difference: 24.9%, p = 0.034) than the synthetic control. We found mixed effects of the opening of PDUs on secondary outcomes. Meta-analyses indicated a significantly lower mean length of stay for psychiatric admissions (-6.4 days, p < 0.001) for patients in mental health trusts with a PDU compared to the synthetic control and no significant effects on other outcomes. Heterogeneity of effect across sites probably reflects variation in PDU configuration and implementation. Further research should explore the intended aims of PDUs alongside how they operate in practice.

**Registration**: The study is registered with the ISRCTN (ISRCTN77588384)

## Introduction

Internationally, acute psychiatric healthcare is in crisis [1–5]. Visits to emergency departments (EDs) for mental health issues are increasing while the number of available psychiatric inpatient beds is decreasing, putting intense pressure on the ED system and causing lengthy waits in ED for people in mental health crisis [6,7]. Approximately two thirds of all people with multiple attendances at ED in England have previously been in receipt of mental health support, suggesting they are not receiving enough support from mental health services to avert mental health crises and/or do not have good alternatives sources of help in a crisis [8]. Compared to people presenting to ED with a physical health complaint, those presenting to ED with psychiatric concerns are over six times more likely to wait longer than 4 hours at the ED [9], and report worse experiences in ED and low levels of patient satisfaction [9–11]

In mental health trusts, pressure on psychiatric wards for beds is intense, with 91% of wards operating above the recommended occupancy rate [12]. Admissions following an acute crisis can be brief (often less than 5 days), despite unclear benefits from short stays on psychiatric wards [13]. Inpatient stays are sometimes detrimental to mental health [14], disproportionately harmful to people from some minority ethnic groups [15] and costly [16]. Furthermore, a substantial proportion of inpatient psychiatric referrals could potentially be avoided [17].

Against this background, some mental health trusts in England have opened Psychiatric Decision Units (PDUs), designed to offer time-limited support (typically between 24 and 72 hours) to people in psychiatric crisis, after which discharge to the community, or admission to an inpatient ward occurs [18]. Discharge to the community may consist of discharge to the care of family with referral and/or signposting to other services, monitoring from a Crisis Resolution and Home Treatment Team (including telephone calls and home visits), or support from the Community Mental Health Team. The substantial gap between the level of support available on a ward and that available in the community is a space which is now occupied by PDUs in some areas. PDUs differ from triage or assessment wards in that admission is voluntary, recliner style chairs are available for sleeping (rather than beds) and the nurse-led spaces offer stabilisation, further assessment and signposting to community services [18]. First time users accessing PDUs in England tend to be referred by ED (42%), or the Crisis and Home Treatment Team (20%). Most (55%-82%) did not have a psychiatric diagnosis, and a high proportion (31%-42%) were previously unknown to mental health services [19]. The population accessing PDUs in the UK tend to be quite young (with an average age in the thirties), with an approximately even gender split. [20] Those discharged from a PDU to inpatient psychiatric care ranged between 13% and 32% across sites in one study. [19,20]

A recent interrupted time series study found an immediate reduction in voluntary psychiatric inpatient admissions following the introduction of a PDU to the crisis care pathway [20]. An international systematic review including 67, 505 participants from six studies across twelve countries indicated that the units deliver significant beneficial effects on several outcomes including a reduction in the duration of emergency department stays (by 164.24 min; 95% CI −261.24 to −67.23 min; p < 0.001) and a reduction to the number of in-patient admissions (odds ratio 0.55, 95% CI 0.43–0.68; p < 0.001) [21]. The systematic review only included studies with a comparison group, and many of the studies employed a pre-post design. These studies may be confounded by temporal effects or regression to the mean [22]. Other studies used a comparison site design, but this design may be confounded due to dissimilarity between sites and temporal differences in other crisis care services around each site. There is a need for research employing methods more robust than other non-randomised designs, including causal analysis methods [23,24]. The present study aimed to evaluate the impact of the addition of PDUs on key outcomes in the psychiatric crisis pathway. We analysed NHS hospital activity data at trusts providing emergency services (acute hospital trusts) and trusts providing secondary mental health services (mental health trusts), including PDUs and inpatient wards, at four geographical locations in England. We employed a synthetic control approach to create a counterfactual or ‘synthetic’ control.

## Methods

### Study design

To evaluate the impact of decision units on outcomes, we needed to understand what the outcomes would have been at the trusts if they had not had a decision unit. Comparing actual outcomes at trusts with a PDU to a hypothetical scenario of what the outcomes would be if the trust didn’t have a PDU is called a counterfactual analysis. This kind of analysis establishes a clear causal link. Without a counterfactual, we wouldn’t know if a change in outcomes was caused by the PDU or something else, such as national trends over time. They type of counterfactual analysis we conducted is a synthetic control analysis. This type of approach is particularly useful for the evaluation of population level health interventions when random assignment is impractical. In this population level approach, entire trusts are considered to have received the ‘intervention’ [23,24]. The ‘treatment’ in this case is the introduction of the PDU to the crisis care pathway; hence this paper refers to both mental health and acute hospital trusts with a PDU in the local crisis care pathway as ‘treated trusts’.

### Setting

Following a national survey to locate and characterise PDUs in England [18], four PDUs at geographically distinct locations in England were selected for the study based on the availability of data for the study covering 24 months preceding and following the opening of the PDUs (suburban London (SWLSG), metropolitan Birmingham (BAS), metropolitan Sheffield (SHSC) and rural Lincolnshire (LP)). There are differences in the aims, staffing and referral routes between these PDUs. All four PDUs aim to reduce psychiatric ED attendances. The PDUs in Birmingham (BAS) and Sheffield (SHSC) also aim to reduce psychiatric ED waits longer than 4 hours. The Birmingham unit (BAS) has only aims related to ED. The three remaining units (SWLSG, SHSC, LP) also aim to reduce psychiatric inpatient admissions. The unit in London (SWLSG) also aims to improve the patient experience. The maximum length of stay on the unit varies from 24 to 72 hours across the units, and the staff: patient ratio ranges from 1:1–1:4 (Table 1). The PDUs launched between November 2014 and March 2019. The period of interest at each site is 24 months preceding and following the opening of the PDU. We analysed data about the local mental health trust (which we refer to as ‘linked mental health trust’) and linked acute hospital trusts. Two of the four mental health trusts in the study had two linked acute hospital trusts, and two had only one (Table 1).

**Table 1:** PDU aims, operating characteristics and linked mental health and acute hospital trusts.

Linked Mental Health Trust (acronym); NHS Trust code, opening date	Aims	Location (Referral Sources), capacity	Maximum Stay	Staff mix on day shift; staff: patient ratio	Linked Mental Health Trust study period	Linked Acute Hospital Trust(s) (acronym), NHS Trust code	Linked Acute Trust(s) study period
South West London & St. George’s Mental Health Trust (SWLSG); RQY, Nov 2016	To reduce psychiatric ED attendances; to reduce psychiatric inpatient admissions; to improve patient experience	Psychiatric hospital (ED, CRHT, Street Triage), 5 (increased to 7 during the COVID-19 pandemic)	48 hours	3 mental health nurses, 3 HCAs, 0.5 psychiatrist, 1 administrator; 1:1 (decreased to 5:7 during the COVID-19 pandemic)	Nov 2014 – Oct 2018	St George’s University Hospitals NHS Foundation Trust (SGUH); RJ7	Excluded – sparse data^§^
						Kingston Hospital NHS Foundation Trust (KH); RAX	Mar 2015 - Oct 2018^§^
Lincolnshire Partnership NHS Foundation Trust (LP); RP7, Jan 2018	To reduce psychiatric ED attendances; to reduce psychiatric inpatient admissions.	Psychiatric hospital (ED, CRHT, Street Triage (16 months after PDU opened), AMHPs), 6	24 hours	1 mental health nurse, 2 HCAs, 0.5 psychiatrist, 0.5 service manager; 1:2	Jan 2016 – Dec 2018^§^	United Lincolnshire Hospitals NHS Foundation Trust (ULH); RWD	Jan 2016 – Dec 2019
Birmingham and Solihull Mental Health Trust (BAS); RXT, Nov 2014	To reduce psychiatric ED attendances; to reduce psychiatric ED waits longer than 4 hours.	Psychiatric hospital (ED, CRHT, Street Triage), 8 (decreased to 5 during the COVID-19 pandemic)	Target 24 hours (initially 72 hours)	1 mental health nurse, 1 HCA, 1 psychiatrist;	Nov 2012 – Oct 2016	Sandwell and West Birmingham NHS Foundation Trust (SWB); RXK	Jun 2013 – Oct 2016^§^
						University Hospitals Birmingham NHS Foundation Trust (UHB); RRK	Nov 2012 - Oct 2016
Sheffield Health and Social Care NHS Foundation Trust (SHSC); TAH, Mar 2019	To reduce psychiatric ED attendances; to reduce psychiatric ED waits longer than 4 hours; to reduce psychiatric inpatient admissions.	Psychiatric hospital on a general hospital site (ED, CRHT, Street Triage, CMHT), 5	48 hours	2 mental health nurses, 1 psychiatrist, 2 support workers, 1 service manager, 1 administrator; 4:5	Excluded – no data^§^	Sheffield Teaching Hospitals NHS Foundation Trust (STH); RHQ	Mar 2017 - Jan 2020^§^

Study periods are typically 24-months pre- and post-PDU opening except where noted. ^§^Trusts or selected months were excluded due to HES lack of submission or recording and quality issues.

AMHP – Approved Mental Health Professional – these professionals can make formal legal decisions regarding whether to detain a person under the Mental Health Act.

CMHT – Community Mental Health Team – a secondary mental health care service composed of a multidisciplinary team operating in the community.

CRHT – Crisis Resolution & Home Treatment team – a team which provides intensive short-term support to service users in crisis at home including telephone calls and visits.

ED – Emergency Department, also known as Accidence and Emergency (A&E) in the UK.

Street Triage – an emergency response service in which mental health professionals provide telephone support or accompany police and paramedics attending disturbances.

HCA – Health Care Assistant.

### Data sources

Patient-level hospital activity data were obtained from Hospital Episode Statistics (HES) admitted patient care (HES-APC) and emergency care (HES-ED) datasets between November 2012 and January 2021 (latest available data at time of study) [25]. This includes data for all study periods spanning 24 months before the first PDU opened in Birmingham in November 2014 and 22 months after the last PDU opened in Sheffield in March 2019 (the follow-up in Sheffield was 22 rather than 24 months to facilitate progress in the data analysis). Each HES record represents a finished consultant episode (FCE) defining a strict period of care for one patient at a single hospital under a single consultant. A continuous spell in hospital can comprise a linked set of FCEs. A continuous spell in ED typically comprises a single FCE. Some mental health trusts submit data to the Mental Health Services data set rather than HES-APC, meaning data from some trusts were unavailable for this study.

Characteristics of acute hospital trusts across a number of dimensions including population deprivation, ED attendance rate and full-time equivalent staff for the financial year 2018/19 were obtained from the NHS Trust Peer Finder Tool [26]. Additional characteristics of acute hospital trusts between 2011 and 2018, including trust catchment population size, were obtained from Public Health England [27]. We aggregated continuous patient spells for service users aged between 18 and 75 years to create 48-month trust-level data series spanning 24 months pre- and post-PDU opening at each trust.

### Outcome measures

#### Mental health trusts.

**Fig 1:**
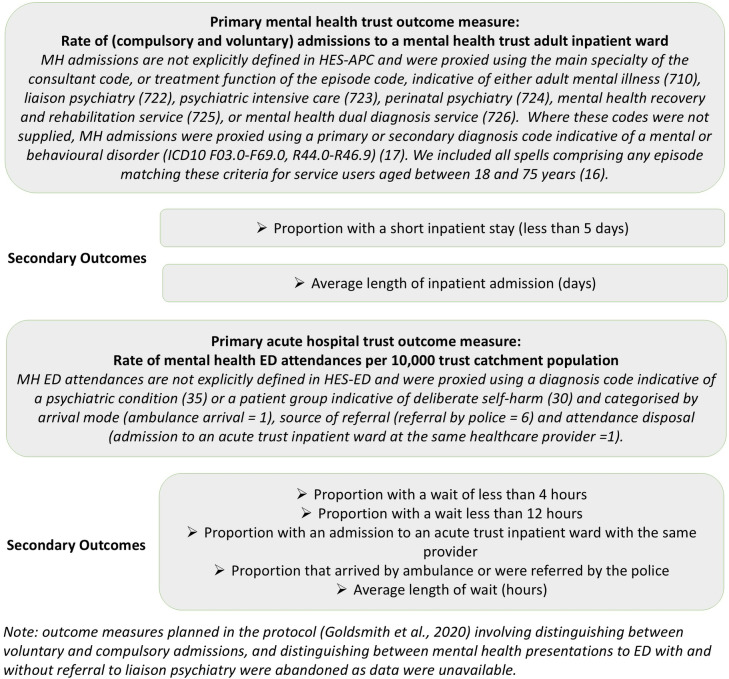
Outcome Measures.

The primary outcome for mental health trusts was the rate of (compulsory and voluntary) admissions to a mental health trust adult inpatient ward per 10,000 trust catchment population (hereafter referred to as MH admissions). The two mental health trust secondary outcomes were the proportion with a short inpatient stay (less than 5 days) and the average length of inpatient admission (measured in days). Outcome measures, alongside additional details about how the primary outcomes were identified in the HES datasets are detailed in Fig 1.

Outcome measures planned in the protocol (Goldsmith et al., 2020) involving distinguishing between voluntary and compulsory admissions, and distinguishing between mental health presentations to ED with and without referral to liaison psychiatry were abandoned as data were unavailable.

#### Acute hospital trusts.

Our primary outcome for acute hospital trusts was the rate of mental health ED attendances per 10,000 acute hospital trust catchment population (hereafter called MH ED attendances). Secondary outcomes were the proportion with a wait of less than 4 and 12 hours; the proportion with an admission to the acute hospital trust inpatient ward at the same provider; the proportion that arrived by ambulance or were referred by the police; and the average length of wait (hours); see Fig 1.

### Statistical analysis

#### Checking data quality.

The data quality of outcomes was assessed by plotting the primary outcomes during the 24 months preceding and 24 months following the opening of the PDU at each trust – both treated trusts and trusts which could potentially be included in the synthetic control. Where necessary due to data sparsity, we shortened the time period for the comparison or excluded trusts from the analysis.

### Creating the synthetic controls and comparing the ‘pre’ periods between treated trusts and synthetic controls

We refer to trusts with a PDU in the local crisis care pathway as ‘treated trusts’. The NHS Trust Peer Finder Tool is designed to match and rank trusts for comparison, determining the similarity based on a range of characteristics. We used data from 2018/19 and methods described in the tool documentation [26] to identify bespoke subsets of trusts which could potentially comprise the synthetic control that were most similar to each treated trust across a range of dimensions, including population deprivation, ED attendance rate and full-time equivalent staff. Importantly, trusts with a PDU in the local crisis care pathway were identified in a mapping study [18] and excluded. The most similar 10 and 20 control trusts to treated mental health and acute trusts respectively were used to create synthetic control groups for each treated trust. Fewer controls were used for mental health trusts as there are fewer mental health trusts. Chi-square tests for no difference between the distribution of key characteristics in the treated trust and their controls in the ‘pre’ period were performed allowing for a Bonferroni correction for multiple testing.

### Analytical covariates

For all outcomes, we adjusted for the size of the trust catchment population. For the primary outcomes i.e., MH ED attendances or MH admissions, we also adjusted for the monthly proportions of the trust catchment population by sex and age. These reflect the characteristics of the population-at-risk, (i.e. the population of the trust catchment area). For the secondary outcomes, the population-at-risk is a subset of the trust catchment population (i.e. only those with a stay on a ward are at risk of a particular duration of inpatient stay). We risk adjusted for the size of the population-at-risk; the proportion of the population-at-risk by sex, age and ethnicity groups and with 2 or more comorbidities according to all their inpatient admissions recorded in HES-ED in the preceding 24 months. Estimates of acute hospital trust catchment population sizes were sourced from Public Health England [27]*.* Estimates of mental health trust catchment population sizes were unavailable. To estimate these, we first fitted a model to predict acute hospital trust catchment population size using trust characteristics in the NHS Trust Peer Finder Tool [26]. This model was then used to predict the catchment population size of each mental health trust based on the same characteristics.

### The generalised synthetic control method

We used the generalised synthetic control (GSC) method [28] to estimate the impact of the PDU opening on each outcome in turn. Essentially, GSC uses mixed effects regression modelling applied to data from a single treated trust and multiple control trusts to estimate a counterfactual, or synthetic control, for each outcome. GSC implicitly assigns weights to each of the trusts in the control group to specify a model that provides the best fit to the outcomes observed in the treated trust in the 2 years before the PDU opened. We refer to this as the synthetic control. The rationale is that the predicted outcomes for the synthetic control during the 2 years after a PDU opened will reflect the outcomes that would have occurred in that specific treated trust if the PDU had not opened. A comparison between treated trust and synthetic control outcomes for the period following the opening of the PDU then provides an estimate of the net effect on outcomes attributable to the PDU. Significance was assessed by a parametric bootstrap procedure [28]. Standard diagnostic checks were performed to test the validity of method assumptions [29]. We used the ‘gsynth’ package in R [30] to implement the GSC method [31]. Bouttell et al. (2018) provides further information on the use of synthetic control methods for evaluating public health interventions [32]. Results were combined in random-effects meta-analyses to generate pooled estimates for outcomes across trusts.

We conducted sensitivity analysis for the trends and approximate size of significant effect estimates by replicating the analyses. For mental health trusts, we used the most similar 20 trusts (as opposed to 10 in the main analysis), and for acute hospital trusts, the most similar 10 (vs most similar 20 in the main analysis). This study is registered with the ISRCTN (ISRCTN77588384), and the statistical analysis protocol is published [33].

### Ethical considerations

The research programme was registered with and received governance approval from research and development (R&D) departments of participating NHS Trusts. Approval for the project was granted from the East Midlands Leicester South Research Ethics Committee (19/EM/0226). This work uses data provided by patients as part of their care and support. Individual patient-level data and data supplied under specific data sharing agreements cannot be made available by the study team. The data were collated, maintained, and quality assured by NHS Digital, now part of NHS England. Requests for access to these data should be directed to the Data Access Request Service, which is part of NHS England (https://digital.nhs.uk/services/data-access-request-service-dars). The data were accessed on April 1st 2021 and there was no ability to identify individual participants at any point.

## Results

### Data quality checks

We first checked the data quality. HES data was available for 41/50 (82%) of mental health trusts in England (using 2018/19 data as an example) and 142/158 (90%) of acute hospital trusts in England (2018/19 data) [26]. No data was found in HES for SHSC, one of the treated mental health trusts, so this mental health trust was excluded. Four mental health trusts and 9 acute hospital trusts were excluded from the pool of trusts from which the synthetic control was drawn as they had a local PDU during the study period [18]. The dataset for the remaining 37 mental health trusts (3 treated and 34 in the ’pool’ of controls) extracted from HES-APC to proxy admissions to a mental health inpatient ward included 725,361 records for service users aged between 18 and 75 years from December 2012 to January 2021. The dataset for the remaining 133 acute hospital trusts (6 treated and 127 in the ‘pool’ of controls) extracted from HES-ED dataset to proxy MH attendances at ED contained 1,775,237 records for service users aged between 18 and 75 years from December 2012 to January 2021.

We plotted the primary outcomes during the 24 months preceding and 24 months following the opening of the PDU at each treated trust and trust which could potentially be included in the synthetic control to assess data quality of outcomes. SGUH, one of the treated acute hospital trusts, was excluded from analysis due to data sparsity of acute hospital trust outcomes. Study periods for LP, KH and STH were shortened due to data sparsity in certain months. Additionally, 3/34 in the pool of control mental health trusts and 12/127 in the pool of control acute hospital trusts were excluded due to data quality. Following exclusions, there were 3 treated mental health trusts, 5 treated acute hospital trusts and 34 and 115 trusts in the pools of control mental health and acute hospital trusts respectively. The trusts comprising the synthetic control for each treated trust are detailed in S1 Table.

### Comparison of mental health trusts to their synthetic controls

In the pre-implementation period, service users admitted to a psychiatric inpatient ward at treated and synthetic control trusts had broadly similar characteristics, including age, sex and length of stay. The population staying on psychiatric wards in the cities served by SWLSG and BAS were more ethnically diverse than their synthetic control trusts – for example service users at SWLSG were more ethnically diverse with 66.1% white ethnicity vs. 77.9% in the controls. There were significant differences in the diagnostic profiles (ICD-10 primary diagnostic code) for service users at SWSLSG compared to the synthetic control. There were significant differences in the method of admission for all three treated trusts when compared to their synthetic controls, including the proportion admitted from Mental Health Crisis Resolution Teams (a team which provides intensive short-term support to service users in crisis at home including telephone calls and visits), which do not exist at all mental health trusts. At BAS, the source of admission to a psychiatric ward differed significantly from the synthetic control trusts, with a smaller proportion of service users admitted from their usual place of residence compared to the synthetic control trusts. At LP, the number of service users with 2 or more Elixhauser comorbidities [34] differed significantly from the synthetic control trusts (S2 Table).

### Comparison of acute hospital trusts to their synthetic controls

In the pre-intervention period, there were no significant differences in age, sex or arrival mode between the five treated trusts and the populations comprising their synthetic controls. There were significant differences in the ethnic makeup of the population at one treated trust (SWB) compared to the trust synthetic control. Three treated trusts (KH, SWB and STH) differed significantly to their synthetic controls in diagnostic codes used at ED. Four treated trusts differed significantly to their synthetic controls in patient group (KH, SWB, UHB and STH), and in referral source (KH, ULH, UHB and STH). All treated trusts differed significantly to their synthetic controls in the discharge method (S3 Table).

### Checking the assumptions required for the GSC method

A minority of outcomes for particular trusts failed diagnostic tests indicating that assumptions required for the GSC method were not satisfied. Consequently, we do not report estimates for these outcomes. This applied to the rate and length of stay of MH admissions in LP, the rate of MH ED attendances in KH, the proportion of MH ED attendances less than 4 hours in UHB, and less than 12 hours in all trusts except UHB and STH. Additionally, we do not report estimates for the proportion of ED attendances that were admitted in KH and ULH due to diagnostic evidence of extrapolation.

### Estimated impacts of PDU opening

The meta-analytic results across sites are reported first, followed by results for individual sites.

### Mental health trust outcomes

There was no significant pooled effect on the primary mental health trust outcome of MH admissions per 10,000 trust catchment patients per month. For the secondary outcomes, there was a significant reduction in the length of MH admissions of -6.4 days, p < 0.001. This meta-analytic result was comprised of a strong, significant reduction in the duration of MH admissions at BAS and a small, non-significant increase at SWLSG. For the secondary outcome, the proportion of short-stay (<5 day) MH admissions, there was overall no significant effect (-0.5%, p = 0.688). At individual sites, there was a significant reduction at SWLSG (-6.5, p < 0.001), a significant increase at BAS (4.6, p = 0.044), and a small, non-significant increase in this outcome at LP (S1 Fig and Table 2)

### Acute hospital trusts

There was no significant pooled effect on the primary outcome of ED MH attendances (per 10,000 trust catchment patients per month). There were no significant pooled effects for any of the secondary outcomes. For individual acute hospital trusts, there was a significant effect at one trust for the following secondary outcomes; at BAS there was a significant reduction in ED MH attendances (per 10,000 trust catchment patients per month) (-1.5%, p = 0.034). At UL, there was a reduction in the proportion of ED MH attendances arrived by police or ambulance (-9.1%, p = 0.003). At KH, the length of wait in ED for MH attendances reduced (-24.6 minutes, p = 0.034). Also at KH, there was a significant increase in the proportion of ED MH attendances with a wait of less than 4 hours (4.0%, p = 0.012). (Table 3 and S2 Fig).

### Sensitivity analysis

In the sensitivity analyses some of the effects lost significance, but the direction and approximate magnitude of effects were robust to changes in the selection of trusts in the synthetic controls.

**Table 2:** Risk-adjusted outcomes for mental health trusts.

MH admissions (per 10,000 trust catchment patients per month)			MH admissions; length of stay (days)			Proportion of MH admissions length of stay < 5 days (%)		
Average valueα	Absolute difference (RD %)β	p-value	Average valueα	Absolute difference (RD %)β	p-value	Average valueα	Absolute difference (RD %)β	p-value
*SWLSG (London) (November 2016 – October 2018)*								
13.8	-0.4 (-2.5)	0.310	57.3	2.6 (4.7)	0.331	**11.0**	**-6.5 (-36.9)**	***<0.001***
*LP (Lincolnshire) (January 2018 – December 2018)*								
32.4	–	–	37.6	–	–	16.4	0.8 (5.4)	0.578
*BAS (Birmingham) (November 2014 – October 2016)*								
17.3	-0.5 (-2.68)	0.749	**73.7**	**-15.5 (-17.3)**	***<0.001***	**12.6**	**4.6 (58.3)**	***0.044***
*Meta-Analysis (24 months* ^*§*^ *post-PDU opening)*								
–	-0.4	0.310	**–**	**-6.4**	***<0.001***	–	-0.5	0.688

α Average value of the outcome in the treated trust. § LP only contributed to the first 12 months in meta-analyses.

β The absolute difference (AD) and relative difference (RD) between the average outcome in the treated trust and the synthetic control.

**Table 3:** Risk-adjusted outcomes for acute hospital trusts.

ED MH attendances (per 10,000 acute hospital trust catchment patients per month)			Proportion of ED MH attendances												ED MH attendance length of wait (minutes)		
			Wait < 4 hours			Wait < 12 hours			Admitted to an acute bed at same provider			Arrived by ambulance or police					
Average value^α^	Absolute difference (RD %)^β^	p-value	Average value^α^	Absolute difference (RD %)^β^	p-value	Average value^α^	Absolute difference (RD %)^β^	p-value	Average value^α^	Absolute difference (RD %)^β^	p-value	Average value^α^	Absolute difference (RD %)^β^	p-value	Average value^α^	Absolute difference (RD %)^β^	p-value
*Kingston Hospital NHS Foundation Trust (KH) (November 2016 – October 2018)*																	
8.6	–	–	99.6	4.0 (4.2)	***0.012***	99.9	–	–	11.5	–	–	46.7	-3.3 (-6.6)	0.957	26.2	-24.6 (-48.5)	***0.034***
*United Lincolnshire Hospitals NHS Foundation Trust (UL) (January 2018 – December 2019)*																	
7.0	0.5 (7.2)	0.510	90.1	-2.4 (-2.6)	0.114	100.0	–	–	7.1	–	–	35.8	-9.1 (-20.4)	***0.003***	121.8	23.6 (24.0)	0.328
*Sandwell and West Birmingham NHS Foundation Trust (SWB) (November 2014 – October 2016)*																	
9.9	2.0 (25.3)	0.146	99.2	0.2 (0.2)	0.307	100.0	–	–	13.1	-1.1 (-8.0)	0.877	58.9	0.4 (0.7)	0.869	82.1	-4.7 –(5.4)	0.360
*University Hospitals Birmingham NHS Foundation Trust (UHB) (November 2012 – October 2016)*																	
3.1	-0.1 (-2.3)	0.904	99.5	–	–	100.0	0.3 (0.3)	0.715	12.9	6.7 (106.2)	0.159	57.3	1.8 (3.3)	0.994	71.5	-2.9 (-3.9)	0.970
*Sheffield Teaching Hospitals NHS Foundation Trust (STH) (March 2019 – January 2020)*																	
4.7	-1.5 (-24.9)	***0.034***	88.2	-1.8 (-2.0)	0.437	99.9	-0.1 (-0.1)	0.843	7.9	-3.6 (-31.3)	0.451	48.8	5.3 (12.3)	0.062	141.7	35.0 (32.9)	0.550
*Meta-Analysis (24 months§ post-PDU opening)*																	
–	-0.2	0.774	–	0.2	0.907	–	0.1	0.580	–	0.0	0.989	–	-1.0	0.723	–	1.1	0.902

α Average value of the outcome in the treated trust. § Sheffield Teaching only contributed to the first 10 months in meta-analyses.

β The absolute difference (AD) and relative difference (RD) between the average outcome in the treated trust and the synthetic control.

## Discussion

### Main Findings

Examining the results at an individual site level reveals some significant effects alongside heterogeneity in the magnitude and direction of effects. In the section below we consider how heterogeneity of PDU configuration and setting within the crisis care pathway might account for our findings at each trust.

### Exploring heterogeneous outcomes

At BAS, we found that the addition of the PDU to the crisis care pathway had no effect on psychiatric inpatient admissions or presentations to ED for mental health problems. The BAS PDU had previously been evaluated by Trethewey et al. [35]. Trethewey found an association between the introduction of the PDU and a reduction in psychiatric inpatient admissions via Liaison Psychiatry (who provide the ED psychiatric service) in Birmingham. Specifically, Trethewey found that the number of patients admitted to a ward via liaison psychiatry reduced from 298 to 219 in the post-period, but did not test for significance. Neither is it clear how many of the patients directed from Liaison Psychiatry to the PDU were discharged to an inpatient ward from the PDU. Trethewey did not identify the impact of the PDU on overall psychiatric inpatient admissions, addressing different outcomes to the current study.

Trethewey also found the introduction of the PDU to be associated with a 39% decrease in the ED psychiatric attendances from the Street Triage team in Birmingham [35]. Street Triage is a mobile mental health service that works with the police, particularly on weekend evenings, to help people displaying mental health problems which are of concern to the police to be met with an appropriate trauma and psychiatrically informed response. We did not examine the effect of the PDU on ED presentations via Street Triage, so it is unclear whether our study would have also found this effect. The introduction of a PDU provides Street Triage with an alternative place to take patients in crisis and without any acute health issues (e.g. without self injury). For these patients, the PDU is likely a more appropriate, calm, and conducive place for their psychiatric crisis to stabilise, away from the busy and noisy ED environment. Features of the configuration of the PDU in Birmingham support this use - the average length of stay at the Birmingham PDU is 4 hours [18], meaning the patient throughput is high so the PDU will often be able to accept additional patients from Street Triage. It is important to note that only a small proportion of patients seen at the PDU in Birmingham have been seen by Street Triage (less than 2%) [19]), so the effect identified by Tretheway may not have the power to have a significant effect on overall ED presentations in Birmingham. Our finding of no impact of the PDU on acute hospital trust ED psychiatric presentations or mental health inpatient admissions across the entire trusts may reflect a lack of change in overall admissions, which does not exclude the possibility of a reduction in admissions from liaison psychiatry.

At BAS, our finding of a significantly shorter MH length of stay with a greater proportion of service users staying for fewer than 5 days compared with the synthetic control runs counter to the expected effect of PDUs, which aim to reduce the proportion of voluntary or short MH admissions. This would have the likely effect of increasing the average length of stays. A large proportion of psychiatric admissions at BAS during the 24 months after the PDU opened may have been admissions that did not come via ED or the PDU (e.g. emergency transfers from another provider or planned admissions), which may have dominated the trust-wide trends in the length of admissions. Additionally, shorter length of stay could be attributable to the effect of other activities or initiatives targeting reductions in length of stay that occurred in the trust at the same time (but were not being introduced in the trusts which make up the control area). Additionally, this could reflect to the specific configuration and focus of this PDU compared with the other PDUs studied. The PDU at BAS has aims solely focussed on psychiatric presentations to ED (to reduce both psychiatric ED attendances and psychiatric ED waits longer than 4 hours). The BAS PDU had higher throughput, shorter PDU stays [20], and a lower staff-to-patient ratios. This configuration may not as readily prevent admissions to acute hospital psychiatric wards that are shorter in length for individuals with less severe presentations.

The proportion of MH admissions with a stay of less than 5 days was significantly lower in SWSLG than the synthetic control suggesting that the PDU may have reduced the need for MH admissions for service users who could be better served by a stay at the PDU. However, we did not observe any significant impact on the overall average length of stay, or the number of MH admissions at SWSLG. This finding is aligned with a recent interrupted time series study which also explored the impact of the addition of a PDU to the crisis care pathway at SWLSG, which found that this trust experienced lower voluntary inpatient admissions in the short term, as well as in the longer-term, with an increase in the share of compulsory service users staying on the ward [20].

We cannot distinguish between the effects of the PDU, which opened in November 2016, and the effects of the other new initiatives in the crisis care pathway at SWSLG and KH. In April 2017 several recovery cafés (informal places where individuals in psychiatric crisis can present with or without carers to access support) opened. In May 2017 a flexible out-of-hours crisis service was also introduced. This offered 7-day street triage as well as home-based assessments and treatments. These initiatives could have contributed to the impacts on the proportion of short stay MH admissions and ED waiting times observed at SWSLG.

We only found significant evidence of a reduction in the rate of MH ED attendances in STH. However, service users in KH waited 24.6 minutes less than the control area leading to significantly fewer 4-hour breaches. Lower rates of MH ED attendances in STH may be a result of the ability of community MH teams to refer directly to the PDU bypassing ED; this referral mechanism was not in place in the other trusts which would not see a reduction in MH ED attendances as most PDU referrals would be made from within ED. At ULH significantly fewer service users arrived at the hospital by ambulance or police compared with the control area. This finding could be attributable to a nurse working in the police control room during the study period to help communicate with people who are experiencing mental health problems and interacting with the police (addressing the crisis and connecting them to community mental health services), rather than to the impact of the PDU.

### Exploring meta-analysis results

Meta-analytic pooling of effect estimates across trusts revealed no significant effects on primary outcomes for either mental health or acute hospital trusts. There was a significant pooled effect on the secondary outcome of length of stay for MH admissions at mental health trusts (-6.4 days, p < 0.001). There were no significant differences in the pooled effects of any of the other secondary outcomes. Pooled analyses address an important NHS policy level question, which is, given the variation in the configuration of PDUs, does the introduction of a PDU typically have an impact on the mental health crisis care pathway? However, interpretation of findings is complicated by potential changes in local conditions outside the hospital settings of each site which may have also impacted observed trends. For example, reduced availability and/or quality of community mental health services have been linked with higher rates of compulsory psychiatric admissions in England [36], and changes in the presence or absence of community support provided outside the NHS may be linked to the rates of local people entering into mental health crisis.

### Comparison with previous studies

A recent international systematic review of psychiatric decision units reported a significant reduction in ward admissions after the opening of a short-stay crisis unit based on four studies. The combined odds ratio was 0.55 (95% CI 0.43 to 0.69) [21]. However, we find no significant impacts of the opening of the PDUs on MH admissions at any of the mental health trusts. The same systematic review found a reduction in the waiting time in ED of –164.24 minutes (95%CI –261.24 to –67.23 minutes), based on two studies reporting results which could be meta-analysed. The present study did not find a significant reduction in waiting times at ED in the pooled results, although ED waiting times were significantly reduced at one trust. This may be connected to the limited capacity of PDUs relative to the size of the ED (the unit capacities range from 5 to 8 service users at any one time; Table 1). It may be that the PDUs in the systematic review were accepting a higher proportion of local service users in mental health crisis, and so had a greater impact on both ward admissions and waiting times in ED. It may be that the EDs in the present study were under so much pressure with staff struggling to meet the demand that they were less effective at rapidly referring suitable patients to the PDU than the EDs linked to the PDUs in the international literature review.

### Strengths and limitations

The synthetic control method offers advantages over other alternative evaluation methods. Firstly, the control is selected using data-driven methods which may reduce researcher bias compared with manual selection. Further it does not rely on an assumption of parallel trends and can control for time varying effects including national changes in policy and trends over each of the 4-year study periods. The generalised synthetic control approach used here has been found to be the most reliable in comparison to alternative approaches [37].

However, comparison of service user characteristics between treated trusts and their synthetic control groups revealed some significant differences highlighting both the difficulty of finding control trusts that are genuinely similar to the treated trusts and of aggregating findings across multiple treated trusts. In general, the synthetic controls for the acute hospital trusts were more similar, this may be accounted for as there are more acute hospital trusts than mental health trusts - offering a greater pool from which the synthetic control can be made.

Predicted outcomes were estimated using control trusts with similar characteristics to the treated trusts across selected variables. We noted some differences between treated and control trusts in the pre-intervention period which may reflect differences in the underlying disease burden, socio-economic and other patient characteristics of the catchment population; or differences in the trust or local healthcare infrastructure, workforce or ways in which healthcare utilisation is coded. These variables are not expected to vary over time and so were not controlled for further in the analysis. Estimates were risk-adjusted for other selected trust and patient-level variables (including ethnicity) to control for a wide variety of unobserved confounders.

Coding in routinely collected data can be less than satisfactory [38] and any changes in coding practices over our long study period can complicate interpretation of trends over time within and across trusts. One site did not contribute data to HES, and some months were excluded from the study periods due to data quality issues in 4 sites; this decreased the power of corresponding analyses and their impact in meta-analyses. The exclusion of certain sites and shortened study periods reduces the risk of potential bias in the study. The limitation of data sparsity is often inherent in ‘real world’ datasets, and studies which do not address them properly (as we have done) risk generating misleading results as their analysis is not robust.

We used a proxy in HES-APC data to capture MH admissions; although, this approach has been verified elsewhere [39] for accuracy by comparison with data on NHS available and occupied beds, estimates may understate the true number of MH admissions in more recent periods [40]. Importantly, this source of data does not distinguish between voluntary and compulsory admissions. As PDUs only accept voluntary patients, any impacts on Trust admissions would primarily be on the number of voluntary admissions. Assessing the impact on all admissions perhaps reduces the power of the analysis.

The impact of changes to other NHS-provided crisis support (such as street triage, crisis cafes and crisis houses), or changes to broader NGO initiatives providing additional alternative sources of help during crises, were not considered here. This could lead to residual unobserved confounding which may lead to bias in our estimates. We were also unable to account for other interventions or local initiatives targeting similar outcomes in control trusts which may dilute the effects observed. Results from meta-analysis for PDUs with different study periods may be confounded by time-varying exposure and background contexts. Additionally, we examined multiple outcomes at multiple sites, each administered without correction for multiple testing. Three results were highly significant (p-value<0.001) but there nevertheless is a risk of Type I errors.

Future research could usefully explore the detail of the choices and decisions people in crisis make about where and from whom they seek support using qualitative or mixed methods studies.

## Conclusions

Heterogenous effects across sites likely reflects the fact that PDUs have been designed and implemented differently in different locations, as part of disparate and changing crisis mental health pathways. Greater clarity of purpose of PDUs might inform future delivery, and further research should focus separately on units that are either aimed at reducing ED attendances or psychiatric admissions to minimise heterogeneity. We found no evidence of any detrimental effect of PDUs on the psychiatric crisis care pathway. PDUs may be preferred by patients and complement other effective inputs into the crisis care pathway such as street triage, crisis cafes, and crisis houses, increasing patient choice, rather than as a sole solution to decreasing inpatient admissions and ED attendances.

## Supporting information

S1 Table Synthetic control trusts for each treated trust, with control trusts ordered by decreasing similarity. (DOCX)

S2 Table Characteristics of service use and service users staying on psychiatric wards at the treated trusts and trusts comprising the synthetic controls in the pre- and post-intervention study periods. (DOCX)

S3 Table Characteristics of service use and service users presenting to emergency departments with mental health complaints in the treated trusts and trusts comprising the synthetic controls in the pre- and post-intervention study periods. (DOCX)

S1 Fig Matrix of graphs showing mental health trust outcomes for treated trusts (red line) and synthetic controls (blue line). (DOCX)

S2 Fig Matrix of graphs showing acute trust outcomes for treated trusts (red lines) and synthetic controls (blue lines). (DOCX)

## References

[1] Anderson EL, Nordstrom K, Wilson MP, Peltzer-Jones JM, Zun L, Ng A, et al. American Association for Emergency Psychiatry Task Force on Medical Clearance of Adults Part I: Introduction, Review and Evidence-Based Guidelines. West J Emerg Med. 2017;18(2):235–42. doi: 10.5811/westjem.2016.10.32258 28210358 PMC5305131

[2] Schmidt M. Frequent visitors at the psychiatric emergency room - A literature review. Psychiatr Q. 2018;89(1):11–32. doi: 10.1007/s11126-017-9509-8 28353131 PMC5807469

[3] Shiraishi M, Ishii T, Kigawa Y, Tayama M, Inoue K, Narita K, et al. Psychiatric Consultations at an Emergency Department in a Metropolitan University Hospital in Northern Japan. Psychiatry Investig. 2018;15(7):739–42. doi: 10.30773/pi.2018.04.04 29945426 PMC6056693

[4] Larkin GL, Beautrais AL, Spirito A, Kirrane BM, Lippmann MJ, Milzman DP. Mental Health and Emergency Medicine: A Research Agenda. Acad Emerg Med 2009;16:1110–1119. 20053230 10.1111/j.1553-2712.2009.00545.x PMC3679662

[5] Wilson MP, Shenvi C, Rives L, Nordstrom K, Schneider S, Gerardi M. Opportunities for Research in Mental Health Emergencies: Executive Summary and Methodology. West J Emerg Med. 2019;20(2):380–5. doi: 10.5811/westjem.2019.1.39260 30881561 PMC6404701

[6] Fleury M-J, Grenier G, Farand L, Ferland F. Use of Emergency Rooms for Mental Health Reasons in Quebec: Barriers and Facilitators. Adm Policy Ment Health. 2019;46(1):18–33. doi: 10.1007/s10488-018-0889-3 30074113

[7] Nicks BA, Manthey DM. The impact of psychiatric patient boarding in emergency departments. Emerg Med Int. 2012;2012:360308. doi: 10.1155/2012/360308 22888437 PMC3408670

[8] Care Quality Commission. Right Here, Right Now: People’s Experiences of Help, Care and Support During a Mental Health Crisis. London: Care Quality Commission; 2015. Available from: https://www.cqc.org.uk/publications/major-report/right-here-right-now-mental-health-crisis-care-review

[9] Care Quality Commission. Key Findings for the National Accident and Emergency Patient Survey. London: Care Quality Commission; 2014. Available from: https://www.cqc.org.uk/sites/default/files/20141201_accident_and_emergency_survey_2014_key_findings.pdf

[10] Care Quality Commission. 2022 Urgent and Emergency Care Survey. London: Care Quality Commission; 2022. Available from: https://www.cqc.org.uk/sites/default/files/2023-07/20230725_uec22_QualityMethodology.odt

[11] Gilburt H. Mental Health under Pressure. London: The King’s Fund; 2015. Available from: https://www.kingsfund.org.uk/insight-and-analysis/reports/mental-health-under-pressure

[12] Crisp N, Smith G, Nicholson K. Old Problems, New Solutions – Improving Acute Psychiatric Care for Adults in England. London: The Commission on Acute Adult Psychiatric Care; 2016. p. 1–6 Available from: https://www.rcpsych.ac.uk/docs/default-source/improving-care/better-mh-policy/policy/policy-old-problems-new-solutions-caapc-report-england.pdf?sfvrsn=7563102e_2

[13] Clibbens N, Harrop D, Blackett S. Early discharge in acute mental health: A rapid literature review. Int J Ment Health Nurs. 2018;27(5):1305–25. doi: 10.1111/inm.12515 29949227

[14] Thibaut B, Dewa LH, Ramtale SC, D’Lima D, Adam S, Ashrafian H, et al. Patient safety in inpatient mental health settings: a systematic review. BMJ Open. 2019;9(12):e030230. doi: 10.1136/bmjopen-2019-030230 31874869 PMC7008434

[15] Morgan C, Mallett R, Hutchinson G, Leff J. Negative pathways to psychiatric care and ethnicity: the bridge between social science and psychiatry. Soc Sci Med. 2004;58(4):739–52. doi: 10.1016/s0277-9536(03)00233-8 14672590

[16] McCrone P, Johnson S, Nolan F, Pilling S, Sandor A, Hoult J, et al. Economic evaluation of a crisis resolution service: a randomised controlled trial. Epidemiol Psichiatr Soc. 2009;18(1):54–8. doi: 10.1017/s1121189x00001469 19378700

[17] Stulz N, Nevely A, Hilpert M, Bielinski D, Spisla C, Maeck L, et al. Referral to Inpatient Treatment Does not Necessarily Imply a Need for Inpatient Treatment. Adm Policy Ment Health. 2015;42(4):474–83. doi: 10.1007/s10488-014-0561-5 24898612

[18] Goldsmith LP, Anderson K, Clarke G, Crowe C, Jarman H, Johnson S, et al. The psychiatric decision unit as an emerging model in mental health crisis care: a national survey in England. Int J Ment Health Nurs. 2021;30(4):955–62. doi: 10.1111/inm.12849 33630402

[19] Goldsmith LP, Anderson K, Clarke G, Crowe C, Jarman H, Johnson S, et al. Service use preceding and following first referral for psychiatric emergency care at a short-stay crisis unit: A cohort study across three cities and one rural area in England. Int J Soc Psychiatry. 2023;69(4):928–41. doi: 10.1177/00207640221142530 36527189 PMC10248300

[20] Smith JG, Anderson K, Clarke G, Crowe C, Goldsmith LP, Jarman H, et al. The effect of psychiatric decision unit services on inpatient admissions and mental health presentations in emergency departments: an interrupted time series analysis from two cities and one rural area in England. Epidemiol Psychiatr Sci. 2024;33:e15. doi: 10.1017/S2045796024000209 38512000 PMC11362677

[21] Anderson K, Goldsmith LP, Lomani J, Ali Z, Clarke G, Crowe C, et al. Short-stay crisis units for mental health patients on crisis care pathways: systematic review and meta-analysis. BJPsych Open. 2022;8(4):e144. doi: 10.1192/bjo.2022.534 35876075 PMC9344431

[22] Torgerson DJ, Torgerson CJ. The Limitations of Before and After Designs. Designing Randomised Trials in Health, Education and the Social Sciences. 2008:9–16. doi: 10.1057/9780230583993_2

[23] Abadie A, Diamond A, Hainmueller J. Synthetic Control Methods for Comparative Case Studies: Estimating the Effect of California’s Tobacco Control Program. J Am Stat Assoc. 2010;105:493–505A.

[24] Abadie A, Diamond A, Hainmueller J. Comparative Politics and the Synthetic Control Method. Am J Pol Sci. 2015;59:495–510.

[25] NHS Digital. Hospital Episode Statistics (HES); Data: NHS Digital [Internet]. 2019. Available from: https://digital.nhs.uk/data-and-information/data-tools-and-services/data-services/hospital-episode-statistics/users-uses-and-access-to-hospital-episode-statistics

[26] NHS Digital. NHS Trust Peer Finder Tool. [software]. 2021 Mar 19. https://digital.nhs.uk/data-and-information/data-tools-and-services/data-services/innovative-uses-of-data/multi-dataset-analysis/nhs-trust-peer-finder-tool

[27] Public Health England NHS Acute (Hospital) Trust Catchment Populations. [software]. 2020 29 Mar. Available from: https://app.powerbi.com/view?r=eyJrIjoiODZmNGQ0YzItZDAwZi00MzFiLWE4NzAtMzVmNTUwMThmMTVlIiwidCI6ImVlNGUxNDk5LTRhMzUtNGIyZS1hZDQ3LTVmM2NmOWRlODY2NiIsImMiOjh9

[28] Xu Y. Generalized Synthetic Control Method: Causal Inference with Interactive Fixed Effects Models. Polit Anal. 2017;25(1):57–76. doi: 10.1017/pan.2016.2

[29] Samartsidis P, Seaman SR, Presanis AM, Hickman M, De Angelis D. Assessing the Causal Effect of Binary Interventions from Observational Panel Data with Few Treated Units. Statist Sci. 2019;34(3). doi: 10.1214/19-sts713

[30] R Foundation for Statistical Computing The R Project for Statistical Computing. [software]. 2003. Available from: https://www.r-project.org/

[31] Xu Y, Liu L. gsynth: Generalized Synthetic Control Method. [software]. 2017. Available from: https://yiqingxu.org/packages/gsynth/.

[32] Bouttell J, Craig P, Lewsey J, Robinson M, Popham F. Synthetic control methodology as a tool for evaluating population-level health interventions. J Epidemiol Community Health. 2018;72(8):673–8. doi: 10.1136/jech-2017-210106 29653993 PMC6204967

[33] Goldsmith LP, Smith JG, Clarke G, Anderson K, Lomani J, Turner K, et al. What is the impact of psychiatric decision units on mental health crisis care pathways? Protocol for an interrupted time series analysis with a synthetic control study. BMC Psychiatry. 2020;20(1):185. doi: 10.1186/s12888-020-02581-5 32326915 PMC7178744

[34] Elixhauser A, Steiner C, Harris DR, Coffey RM. Comorbidity measures for use with administrative data. Med Care. 1998;36(1):8–27. doi: 10.1097/00005650-199801000-00004 9431328

[35] Trethewey SP, Deepak S, Saad S, Hughes E, Tadros G. Evaluation of the Psychiatric Decisions Unit (PDU): effect on emergency department presentations and psychiatric inpatient admissions. Postgrad Med J. 2019;95(1119):6–11. doi: 10.1136/postgradmedj-2018-135788 30765421

[36] Sheridan Rains L, Weich S, Maddock C, Smith S, Keown P, Crepaz-Keay D, et al. Understanding increasing rates of psychiatric hospital detentions in England: development and preliminary testing of an explanatory model. BJPsych Open. 2020;6(5):e88. doi: 10.1192/bjo.2020.64 32792034 PMC7453796

[37] Clarke GM, Steventon A, O’Neill S. A comparison of synthetic control approaches for the evaluation of policy interventions using observational data: Evaluating the impact of redesigning urgent and emergency care in Northumberland. Health Serv Res. 2023;58(2):445–57. doi: 10.1111/1475-6773.14126 36573610 PMC10012235

[38] Davis KAS, Sudlow CLM, Hotopf M. Can mental health diagnoses in administrative data be used for research? A systematic review of the accuracy of routinely collected diagnoses. BMC Psychiatry. 2016;16:263. doi: 10.1186/s12888-016-0963-x 27455845 PMC4960739

[39] The Strategy Unit. Exploring Mental Health Inpatient Capacity across Sustainability and Transformation Partnerships in England. London: The Strategy Unit; 2019. Available from: https://www.strategyunitwm.nhs.uk/publications/exploring-mental-health-inpatient-capacity

[40] NHS Digital. KHO3 Quarterly Bed Availability and Occupancy. 2022. [cited 2023 29 Mar]. Available from: https://digital.nhs.uk/data-and-information/data-collections-and-data-sets/data-collections/kho3-quarterly-bed-availability-and-occupancy

