# Supplementary material for: The impact of psychiatric decision units on mental health crisis care pathways: a synthetic control study
Source: PLOS Ment Health. 2025 May 2;2(5):e0000171. doi: 10.1371/journal.pmen.0000171 (PMC12798399; doi:10.1371/journal.pmen.0000171)
Supplement: S1 Table — (DOCX) [file pmen.0000171.s001.docx]

**S1 Table: Synthetic control trusts for each treated trust, with control trusts ordered by decreasing similarity.**

| **Treated trust code** | **Similarity** | **Control**  **trust code** | **Peer name** | **Treated trust code** | **Similarity** | **Control**  **trust code** | **Peer name** |
| --- | --- | --- | --- | --- | --- | --- | --- |
| **Kingston Hospital NHS Foundation Trust** | | | | **United Lincolnshire Hospitals NHS Trust** | | | |
| RAX | 1 | R1K | London North West Healthcare NHS Trust | RWD | 1 | RCB | York Teaching Hospital NHS Foundation Trust |
| RAX | 2 | RAJ | Southend University Hospital NHS Foundation Trust | RWD | 2 | RD1 | Royal United Hospital Bath NHS Trust |
| RAX | 3 | RD1 | Royal United Hospital Bath NHS Trust | RWD | 3 | RDE | Colchester Hospital University NHS Foundation Trust |
| RAX | 4 | RD3 | Poole Hospital NHS Foundation Trust | RWD | 4 | RGN | Peterborough and Stamford Hospitals NHS Foundation Trust |
| RAX | 5 | RD8 | Milton Keynes Hospital NHS Foundation Trust | RWD | 5 | RGR | West Suffolk NHS Foundation Trust |
| RAX | 6 | RDD | Basildon and Thurrock University Hospitals NHS Foundation Trust | RWD | 6 | RH8 | Royal Devon and Exeter NHS Foundation Trust |
| RAX | 7 | RDU | Frimley Park Hospital NHS Foundation Trust | RWD | 7 | RJL | Northern Lincolnshire and Goole Hospitals NHS Foundation Trust |
| RAX | 8 | RHW | Royal Berkshire NHS Foundation Trust | RWD | 8 | RJR | Countess of Chester Hospital NHS Foundation Trust |
| RAX | 9 | RJ1 | Guy's and St Thomas' NHS Foundation Trust | RWD | 9 | RK5 | Sherwood Forest Hospitals NHS Foundation Trust |
| RAX | 10 | RJR | Countess of Chester Hospital NHS Foundation Trust | RWD | 10 | RLQ | Wye Valley NHS Trust |
| RAX | 11 | RN3 | Great Western Hospitals NHS Foundation Trust | RWD | 11 | RN3 | Great Western Hospitals NHS Foundation Trust |
| RAX | 12 | RN7 | Dartford and Gravesham NHS Trust | RWD | 12 | RN5 | Hampshire Hospitals NHS Foundation Trust |
| RAX | 13 | RPA | Medway NHS Foundation Trust | RWD | 13 | RNS | Northampton General Hospital NHS Trust |
| RAX | 14 | RQ8 | Mid Essex Hospital Services NHS Trust | RWD | 14 | RP5 | Doncaster and Bassetlaw Hospitals NHS Foundation Trust |
| RAX | 15 | RRV | University College London Hospitals NHS Foundation Trust | RWD | 15 | RQ8 | Mid Essex Hospital Services NHS Trust |
| RAX | 16 | RTK | Ashford and St. Peter's Hospitals NHS Foundation Trust | RWD | 16 | RWA | Hull and East Yorkshire Hospitals NHS Trust |
| RAX | 17 | RVR | Epsom and St Helier University Hospitals NHS Trust | RWD | 17 | RX1 | Nottingham University Hospitals NHS Trust |
| RAX | 18 | RWF | Maidstone and Tunbridge Wells NHS Trust | RWD | 18 | RXF | Mid Yorkshire Hospitals NHS Trust |
| RAX | 19 | RWJ | Stockport NHS Foundation Trust | RWD | 19 | RXP | County Durham and Darlington NHS Foundation Trust |
| RAX | 20 | RXQ | Buckinghamshire Healthcare NHS Trust | RWD | 20 | RXW | Shrewsbury and Telford Hospital NHS Trust |
| **Sandwell And West Birmingham Hospitals NHS Trust** | | | | **University Hospitals Birmingham NHS Foundation Trust** | | | |
| RXK | 1 | RBK | Walsall Healthcare NHS Trust | RRK | 1 | R1H | Barts Health NHS Trust |
| RXK | 2 | RBL | Wirral University Teaching Hospital NHS Foundation Trust | RRK | 2 | RCB | York Teaching Hospital NHS Foundation Trust |
| RXK | 3 | REM | Aintree University Hospital NHS Foundation Trust | RRK | 3 | RDE | Colchester Hospital University NHS Foundation Trust |
| RXK | 4 | RFR | The Rotherham NHS Foundation Trust | RRK | 4 | RDU | Frimley Park Hospital NHS Foundation Trust |
| RXK | 5 | RJ2 | Lewisham Healthcare NHS Trust | RRK | 5 | REF | Royal Cornwall Hospitals NHS Trust |
| RXK | 6 | RK5 | Sherwood Forest Hospitals NHS Foundation Trust | RRK | 6 | RGN | Peterborough and Stamford Hospitals NHS Foundation Trust |
| RXK | 7 | RL4 | The Royal Wolverhampton NHS Trust | RRK | 7 | RH8 | Royal Devon and Exeter NHS Foundation Trust |
| RXK | 8 | RLN | City Hospitals Sunderland NHS Foundation Trust | RRK | 8 | RHM | University Hospital Southampton NHS Foundation Trust |
| RXK | 9 | RMC | Bolton NHS Foundation Trust | RRK | 9 | RHU | Portsmouth Hospitals NHS Trust |
| RXK | 10 | RMP | Tameside Hospital NHS Foundation Trust | RRK | 10 | RJE | University Hospital of North Staffordshire NHS Trust |
| RXK | 11 | RNA | The Dudley Group NHS Foundation Trust | RRK | 11 | RL4 | The Royal Wolverhampton NHS Trust |
| RXK | 12 | RNQ | Kettering General Hospital NHS Foundation Trust | RRK | 12 | RN5 | Hampshire Hospitals NHS Foundation Trust |
| RXK | 13 | RP5 | Doncaster and Bassetlaw Hospitals NHS Foundation Trust | RRK | 13 | RTD | The Newcastle Upon Tyne Hospitals NHS Foundation Trust |
| RXK | 14 | RQ6 | Royal Liverpool and Broadgreen University Hospitals NHS Trust | RRK | 14 | RTG | Derby Hospitals NHS Foundation Trust |
| RXK | 15 | RRF | Wrightington, Wigan and Leigh NHS Foundation Trust | RRK | 15 | RTH | Oxford University Hospitals NHS Trust |
| RXK | 16 | RWW | Warrington and Halton Hospitals NHS Foundation Trust | RRK | 16 | RTR | South Tees Hospitals NHS Foundation Trust |
| RXK | 17 | RWY | Calderdale and Huddersfield NHS Foundation Trust | RRK | 17 | RVJ | North Bristol NHS Trust |
| RXK | 18 | RX1 | Nottingham University Hospitals NHS Trust | RRK | 18 | RWE | University Hospitals of Leicester NHS Trust |
| RXK | 19 | RXF | Mid Yorkshire Hospitals NHS Trust | RRK | 19 | RX1 | Nottingham University Hospitals NHS Trust |
| RXK | 20 | RXP | County Durham and Darlington NHS Foundation Trust | RRK | 20 | RXF | Mid Yorkshire Hospitals NHS Trust |
| **Sheffield Teaching Hospitals NHS Foundation Trust** | | | | **South West London and St George's Mental Health NHS Trust** | | | |
| RHQ | 1 | R1H | Barts Health NHS Trust | RQY | 1 | RDY | Dorset Healthcare University NHS Foundation Trust |
| RHQ | 2 | RBN | St Helens and Knowsley Hospitals NHS Trust | RQY | 2 | RH5 | Somerset Partnership NHS Foundation Trust |
| RHQ | 3 | RCB | York Teaching Hospital NHS Foundation Trust | RQY | 3 | RJ8 | Cornwall Partnership NHS Foundation Trust |
| RHQ | 4 | RDE | Colchester Hospital University NHS Foundation Trust | RQY | 4 | RRE | South Staffordshire And Shropshire Healthcare NHS Foundation Trust |
| RHQ | 5 | RGN | Peterborough and Stamford Hospitals NHS Foundation Trust | RQY | 5 | RT5 | Leicestershire Partnership NHS Trust |
| RHQ | 6 | RGT | Cambridge University Hospitals NHS Foundation Trust | RQY | 6 | RTV | 5 Boroughs Partnership NHS Foundation Trust |
| RHQ | 7 | RHU | Portsmouth Hospitals NHS Trust | RQY | 7 | RV3 | Central and North West London NHS Foundation Trust |
| RHQ | 8 | RJE | University Hospital of North Staffordshire NHS Trust | RQY | 8 | RW1 | Southern Health NHS Foundation Trust |
| RHQ | 9 | RK5 | Sherwood Forest Hospitals NHS Foundation Trust | RQY | 9 | RW4 | Mersey Care NHS Trust |
| RHQ | 10 | RL4 | The Royal Wolverhampton NHS Trust | RQY | 10 | RXG | South West Yorkshire Partnership NHS Foundation Trust |
| RHQ | 11 | RN5 | Hampshire Hospitals NHS Foundation Trust | **Lincolnshire Partnership NHS Foundation Trust** | | | |
| RHQ | 12 | RNS | Northampton General Hospital NHS Trust | RP7 | 1 | RLY | North Staffordshire Combined Healthcare NHS Trust |
| RHQ | 13 | RP5 | Doncaster and Bassetlaw Hospitals NHS Foundation Trust | RP7 | 2 | RMY | Norfolk And Suffolk NHS Foundation Trust |
| RHQ | 14 | RRF | Wrightington, Wigan and Leigh NHS Foundation Trust | RP7 | 3 | RNN | Cumbria Partnership NHS Foundation Trust |
| RHQ | 15 | RTG | Derby Hospitals NHS Foundation Trust | RP7 | 4 | RNU | Oxford Health NHS Foundation Trust |
| RHQ | 16 | RVJ | North Bristol NHS Trust | RP7 | 5 | RTQ | 2Gether NHS Foundation Trust |
| RHQ | 17 | RWA | Hull and East Yorkshire Hospitals NHS Trust | RP7 | 6 | RV9 | Humber NHS Foundation Trust |
| RHQ | 18 | RWJ | Stockport NHS Foundation Trust | RP7 | 7 | RVN | Avon And Wiltshire Mental Health Partnership NHS Trust |
| RHQ | 19 | RX1 | Nottingham University Hospitals NHS Trust | RP7 | 8 | RXA | Cheshire And Wirral Partnership NHS Foundation Trust |
| RHQ | 20 | RXF | Mid Yorkshire Hospitals NHS Trust | RP7 | 9 | RXE | Rotherham Doncaster And South Humber NHS Foundation Trust |
|  | | | | RP7 | 10 | RXM | Derbyshire Healthcare NHS Foundation Trust |
| **Birmingham And Solihull Mental Health NHS Foundation Trust** | | | | | | | |
| RXT | 1 | RAT | North East London NHS Foundation Trust | RXT | 6 | RT5 | Leicestershire Partnership NHS Trust |
| RXT | 2 | RH5 | Somerset Partnership NHS Foundation Trust | RXT | 7 | RTV | 5 Boroughs Partnership NHS Foundation Trust |
| RXT | 3 | RHA | Nottinghamshire Healthcare NHS Trust | RXT | 8 | RXA | Cheshire And Wirral Partnership NHS Foundation Trust |
| RXT | 4 | RJ8 | Cornwall Partnership NHS Foundation Trust | RXT | 9 | RXG | South West Yorkshire Partnership NHS Foundation Trust |
| RXT | 5 | RNN | Cumbria Partnership NHS Foundation Trust | RXT | 10 | RXM | Derbyshire Healthcare NHS Foundation Trust |
