# Supplementary material for: The impact of psychiatric decision units on mental health crisis care pathways: a synthetic control study
Source: PLOS Ment Health. 2025 May 2;2(5):e0000171. doi: 10.1371/journal.pmen.0000171 (PMC12798399; doi:10.1371/journal.pmen.0000171)
Supplement: S2 Table — (DOCX) [file pmen.0000171.s002.docx]

**S2 Table: Characteristics of service use and service users staying on psychiatric wards at the treated trusts and trusts comprising the synthetic controls in the pre- and post-intervention study periods. ^Ϯ^**

| Trust | | SWLSG (London) | | | | | LP (Lincolnshire) | | | | BAS (Birmingham) | | | |  |
| --- | --- | --- | --- | --- | --- | --- | --- | --- | --- | --- | --- | --- | --- | --- | --- |
|  | | Pre-PDU | | | Post-PDU | | Pre-PDU | | Post-PDU | | Pre-PDU | | Post-PDU | |  |
|  | | Nov14-Oct16 | | | Nov16-Oct18 | | Jan 16-Dec17 | | Jan18-Dec18 | | Nov12-Oct14 | | Nov14-Oct16 | |  |
|  | | Treated | Control**^ξ^** | | Treated | Control**^ξ^** | Treated | Control**^ξ^** | Treated | Control**^ξ^** | Treated | Control**^ξ^** | Treated | Control**^ξ^** |  |
| N | | 3864 | 39007 | | 3692 | 37557 | 1532 | 29500 | 994 | 30037 | 4602 | 33370 | 4532 | 30919 |  |
| Average admissions per trust per month | | 161 | 163 | | 154 | 156 | 64 | 123 | 83 | 250 | 192 | 139 | 189 | 129 |  |
| Sex | |  |  | |  |  |  |  |  |  |  |  |  |  |  |
|  | Male | 48.9 | 53 | | 53.2 | 52.6 | 56.7 | 51.7 | 55.6 | 51.2 | 58.9 | 54.6 | 54.7 | 53.8 |  |
|  | Female | 51.1 | 47 | | 46.8 | 47.4 | 43.3 | 48.3 | 44.4 | 48.8 | 41.1 | 45.4 | 45.3 | 46.2 |  |
| Age (years) | |  |  | |  |  |  |  |  |  |  |  |  |  |  |
|  | 18 – 24 | 16 | 14.3 | | 15.9 | 15 | 14.8 | 14 | 14.9 | 14.7 | 14.9 | 13.1 | 12.6 | 14.3 |  |
|  | 25-64 | 76.4 | 76.3 | | 77 | 75.5 | 69.5 | 75.1 | 71.2 | 74.7 | 77.5 | 76.5 | 79.3 | 74.9 |  |
|  | 65-74 | 7.6 | 9.4 | | 7.1 | 9.5 | 15.7 | 10.9 | 13.9 | 10.6 | 7.6 | 10.5 | 8.1 | 10.8 |  |
| Ethnicity | |  |  | |  |  |  |  |  |  |  |  |  |  |  |
|  | Asian | 8.4 | 5.1^§^ | | 9.4 | 4.7 | <1 | 1.9 | <1.5 | 2.3 | 17.7 | 5.6^§^ | 16.3 | 5.7 |  |
|  | Black | 16.1 | 4.6 | | 16.1 | 5.1 | <1 | 2.5 | <1.5 | 2.4 | 16.2 | 4 | 15.5 | 4.6 |  |
|  | White | 66.1 | 77.9 | | 63 | 75.7 | 90.2 | 85.3 | 86.8 | 84.8 | 54.5 | 85.4 | 56.8 | 82.5 |  |
|  | Mixed | 3.3 | 2.1 | | 4 | 2.3 | 1.6 | 1.9 | 1.4 | 2 | 4.8 | 1.8 | 4.9 | 1.9 |  |
|  | Other | 6.1 | 10.3 | | 7.5 | 12.2 | 7 | 8.4 | 10.2 | 8.5 | 6.8 | 3.2 | 6.5 | 5.3 |  |
| ICD10 primary diagnosis code | |  |  | |  |  |  |  |  |  |  |  |  |  |  |
|  | F10-19: Mental & behavioural psychoactive substance abuse | 7.7 | 12.2^§^ | | 6.4 | 9.4 | 8.9 | 8.9 | 8.5 | 7.9 | 5.7 | 12.5 | 5.8 | 12.9 |  |
|  | F20-29: Schizophrenia, schizotypal and delusional disorders | 38.3 | 24.5 | | 41.5 | 24.7 | 26 | 23.8 | 17.1 | 22.7 | 40.2 | 24.1 | 36.3 | 25.1 |  |
|  | F30-39: Mood (affective) disorders | 21.4 | 19.3 | | 23.7 | 19.5 | 23.4 | 19.3 | 15.3 | 18.5 | 24.6 | 22.3 | 25.6 | 22.3 |  |
|  | F40-F48: Neurotic, stress-related and somatoform disorders | 7.1 | 6.5 | | 6 | 6.7 | 11.9 | 7.3 | 6.8 | 7.8 | 4.8 | 7.8 | 6.7 | 8.1 |  |
|  | F60-69: Disorders of adult personality and behaviour | 14.6 | 10.7 | | 15.2 | 12 | 16.3 | 13.2 | 11.8 | 13.9 | 8.3 | 10.4 | 11.1 | 12.2 |  |
|  | Other | 10.9 | 26.8 | | 7.2 | 27.7 | 13.4 | 27.5 | 40.5 | 29.2 | 16.5 | 23 | 14.6 | 19.4 |  |
| Source of admission | |  |  | |  |  |  |  |  |  |  |  |  |  |  |
|  | Usual place of residence | 44.5 | 61.3 | | 41.9 | 56.2 | 66.4 | 56.4 | 56.7 | 56.2 | 47.1 | 66.6^§^ | 40.6 | 64 |  |
|  | Temporary place of residence | 3.5 | 2.9 | | 4.4 | 3 | <1 | 3.3 | 2.3 | 2.9 | 2.9 | 2.1 | 2.8 | 2.6 |  |
|  | Penal establishment | 7 | 5.5 | | 6.1 | 4.9 | <1 | 5.1 | 3.6 | 4.2 | 16.1 | 4.3 | 27.8 | 4.2 |  |
|  | NHS other hospital – general ward or A&E | 20.7 | 16.7 | | 25.8 | 20 | 12.3 | 15.6 | 10.2 | 15.8 | 22.6 | 15.9 | 20.5 | 13.3 |  |
|  | NHS other hospital – mental health ward | 15.3 | 8.1 | | 13.4 | 8.2 | 5.5 | 7.3 | 13.8 | 8 | 6.2 | 6.2 | 4.3 | 6.9 |  |
|  | Other | 9 | 5.5 | | 8.3 | 7.7 | 14.3 | 12.3 | 13.4 | 12.9 | 5.2 | 4.8 | 3.9 | 8.8 |  |
| Method of admission | |  |  | |  |  |  |  |  |  |  |  |  |  |  |
|  | Booked | 14.7 | 6.2^§^ | | 9.5 | 5.5 | <1 | 6.4^§^ | <1 | 8.6 | 7.5 | 7^§^ | 0 | 7.8 |  |
|  | Planned (when part of a sequence of clinical care) | 22.1 | 17.5 | | 23.7 | 19.3 | 5.9 | 12.7 | 2.3 | 10.4 | 24.8 | 19.7 | 2.4 | 15.9 |  |
|  | Consultant clinic | 6.8 | 6.8 | | <1 | 3.1 | 1.6 | 8.9 | 1.5 | 7.9 | 1.1 | 13 | 11 | 6.4 |  |
|  | Mental Health Crisis Resolution Team | 29.2 | 15.3 | | 17.5 | 25.9 | 65.2 | 23.7 | 59.8 | 25 | 0 | 5 | 0 | 16.9 |  |
|  | ED or emergency admission transfer from another provider | <1 | 24.6 | | 33.4 | 19.8 | <1 | 12.2 | <1 | 10.8 | 6.5 | 39.6 | 0 | 22.2 |  |
|  | Other | 26.9 | 34 | | 15.9 | 26.4 | 27 | 36.1 | 36.2 | 37.3 | 60.1 | 15.7 | 86.6 | 30.8 |  |
| Length of stay | |  |  | |  |  |  |  |  |  |  |  |  |  |  |
|  | Less than 5 days | 15.8 | 20.3 | | 11.1 | 19.5 | 10.9 | 16.9 | 16.9 | 24.9 | 12.4 | 18.1 | 12.6 | 16 |  |
|  | 5 days or longer | 84.2 | 79.7 | | 88.9 | 80.5 | 89.1 | 83.1 | 83.1 | 75.1 | 87.6 | 81.9 | 87.4 | 84 |  |
|  | No discharge date | 3.4 | 0.7 | | 0.8 | 1.1 | 1.5 | 1.7 | 4.9 | 2.2 | 1.3 | 3.8 | 1.5 | 1.8 |  |
| Number of Elixhauser comorbidities (Elixhauser et al., 1998) | |  |  | |  |  |  |  |  |  |  |  |  |  |  |
|  | < 2 | 61.5 | 64.2 | | 48.6 | 66.2 | 55.5 | 74.8^§^ | 67.3 | 72.4 | 74.2 | 70.8 | 75.8 | 63.1 |  |
|  | >= 2 | 38.5 | 35.8 | | 51.4 | 33.8 | 44.5 | 25.2 | 32.7 | 27.6 | 25.8 | 29.2 | 24.2 | 36.9 |  |
| Note: selected pairs of results were rounded to protect patient confidentiality where group totals were small.  **^Ϯ^** Sheffield Health and Social Care NHS Foundation Trust was excluded as there was no data submitted to HES-ED. | | | | ^§^ Group characteristic failed chi-square test of no difference between the treated trust and the synthetic controls in the pre-implementation period. Only significant differences in the pre-period are noted  **^ξ^** The synthetic control trusts were weighted composites of the control trusts. | | | | | | | | | | | |
