# Supplementary material for: The impact of psychiatric decision units on mental health crisis care pathways: a synthetic control study
Source: PLOS Ment Health. 2025 May 2;2(5):e0000171. doi: 10.1371/journal.pmen.0000171 (PMC12798399; doi:10.1371/journal.pmen.0000171)
Supplement: S3 Table — (DOCX) [file pmen.0000171.s003.docx]

**S3 Table: Characteristics of service use and service users presenting to emergency departments with mental health complaints in the treated trusts and trusts comprising the synthetic controls in the pre- and post-intervention study periods.^Ϯ^**

| Trust | | KH (London) | | | | ULH (Lincolnshire) | | | | | SWB (Birmingham) | | | | UHB (Birmingham) | | | | | | STH (Sheffield) | | | | | | |  |
| --- | --- | --- | --- | --- | --- | --- | --- | --- | --- | --- | --- | --- | --- | --- | --- | --- | --- | --- | --- | --- | --- | --- | --- | --- | --- | --- | --- | --- |
|  | | Pre-PDU | | Post-PDU | | Pre-PDU | | | Post-PDU | | Pre-PDU | | Post-PDU | | Pre-PDU | | | Post-PDU | | | Pre-PDU | | | Post-PDU | | | |  |
|  | | Mar15-Oct16 | | Nov16-Oct18 | | Jan16-Dec17 | | | Jan18-Dec19 | | Jun13-Oct14 | | Nov14-Oct16 | | Nov12-Oct14 | | | Nov14-Oct16 | | | Mar17-Feb19 | | | Mar19-Jan20 | | | |  |
|  | | Treated | Control**^ξ^** | Treated | Control**^ξ^** | Treated | Control**^ξ^** | | Treated | Control**^ξ^** | Treated | Control**^ξ^** | Treated | Control**^ξ^** | | Treated | Control**^ξ^** | | Treated | Control**^ξ^** | | Treated | Control**^ξ^** | | Treated | Control**^ξ^** | | |
| N | | 2,245 | 66,743 | 4,417 | 69,023 | 6,106 | 83,538 | | 5,983 | 93,641 | 5,331 | 78,498 | 7,583 | 79,586 | | 5788 | 73338 | | 6349 | 78531 | | 6486 | 98596 | | 2928 | 53085 | | |
| Sex | |  |  |  |  |  |  | |  |  |  |  |  |  | |  |  | |  |  | |  |  | |  |  | | |
|  | Male | 40.8 | 48.7 | 45.0 | 48.4 | 51.9 | 47.5 | | 49.2 | 48.6 | 59.6 | 52.6 | 58.4 | 50.9 | | 241 | 153 | | 265 | 164 | | 270 | 205 | | 266 | 241 | | |
|  | Female | 59.2 | 51.3 | 55.0 | 51.6 | 48.1 | 52.5 | | 50.8 | 51.4 | 40.4 | 47.4 | 41.6 | 49.1 | | 51.0 | 48.4 | | 47.1 | 47.2 | | 46.9 | 48.5 | | 47.3 | 48.8 | | |
| Age (years) | |  |  |  |  |  |  | |  |  |  |  |  |  | |  |  | |  |  | |  |  | |  |  | | |
|  | 18 – 24 | 28.9 | 24.4 | 26.4 | 24.7 | 28.6 | 26.4 | | 24.9 | 25.7 | 19.4 | 23.5 | 20.2 | 24.8 | | 25.0 | 25.7 | | 28.0 | 26.6 | | 25.2 | 26.3 | | 26.1 | 25.0 | | |
|  | 25-64 | 64.5 | 71.9 | 67.5 | 70.9 | 67.1 | 69.0 | | 66.5 | 67.2 | 78.8 | 73.3 | 77.6 | 71.4 | | 71.9 | 70.3 | | 68.5 | 69.1 | | <70 | 68.0 | | 68.8 | 68.0 | | |
|  | 65-74 | 6.6 | 3.7 | 6.1 | 4.4 | 4.3 | 4.6 | | 8.6 | 7.1 | 1.8 | 3.2 | 2.2 | 3.8 | | 3.2 | 4.1 | | 3.5 | 4.3 | | <6 | 5.8 | | 5.1 | 7.0 | | |
| Ethnicity | |  |  |  |  |  |  | |  |  |  |  |  |  | |  |  | |  |  | |  |  | |  |  | | |
|  | Asian | 7.1 | 5.4 | 7.0 | 5.0 | <1 | 1.5 | | <1 | 1.7 | 17.0 | 3.0 ^§^ | 15.6 | 2.7 | | 8.0 | 3.0 | | 7.9 | 2.4 | | <5 | 3.5 | | <5 | 3.5 | | |
|  | Black | 2.5 | 4.1 | 3.3 | 4.0 | <1 | 0.7 | | <1 | 1.1 | 10.8 | 1.3 | 11.8 | 1.1 | | 4.0 | 1.1 | | 3.8 | 0.8 | | <4 | 1.8 | | <4 | 2.0 | | |
|  | White | 77.7 | 74.8 | 73.5 | 73.4 | 83.8 | 85.5 | | 82.4 | 81.3 | 56.4 | 80.6 | 57.8 | 83.7 | | 70.7 | 85.4 | | 69.9 | 85.0 | | 76.7 | 78.0 | | 78.8 | 74.5 | | |
|  | Mixed | 1.8 | 1.1 | 2.1 | 1.2 | 0.8 | 0.9 | | 1.2 | 1.0 | 2.6 | 1.0 | 2.2 | 1.1 | | 2.4 | 0.9 | | 3.2 | 0.9 | | 2.0 | 1.2 | | 1.5 | 1.2 | | |
|  | Other | 10.9 | 14.5 | 14.1 | 16.4 | 14.9 | 11.4 | | 15.6 | 14.9 | 13.2 | 14.1 | 12.6 | 11.5 | | 14.9 | 9.7 | | 15.1 | 10.8 | | 13.0 | 15.5 | | 12.1 | 18.8 | | |
| ED diagnosis code | |  |  |  |  |  |  | |  |  |  |  |  |  | |  |  | |  |  | |  |  | |  |  | | |
|  | 01:Laceration (01) | <1 | 1.7 ^§^ | 2.8 | 2.2 | 5.2 | 4.6 | | 1.4 | 5.2 | 1.1 | 2.7 ^§^ | 1.2 | 3.0 | | 3.4 | 4.0 ^§^ | | 2.7 | 4.5 | | <3 | 4.4 ^§^ | | 1.3 | 4.4 | | |
|  | 02:Contusion/abrasion | <1 | <1 | 1.1 | 0.6 | 0.9 | 0.7 | | <1 | 2.1 | <0.5 | 0.5 | <0.5 | 0.4 | | <0.5 | 0.5 | | <1 | 0.5 | | <2 | 1.3 | | <1 | 1.9 | | |
|  | 05: Dislocation/fracture/joint injury/amputation | <1 | <1 | 1.3 | 0.6 | 1.2 | 0.6 | | <1 | 1.9 | <0.5 | 0.4 | <0.5 | 0.3 | | 0.5 | 0.3 | | 1.0 | 0.4 | | <2 | 1.3 | | <0.5 | 1.8 | | |
|  | 14:Poisoning(incl. overdose) | <5 | 8.9 | 4.8 | 7.3 | 21.5 | 19.2 | | 2.7 | 14.6 | 7.4 | 13.9 | 5.1 | 16.1 | | 15.3 | 19.6 | | <15 | 18.0 | | <10 | 13.5 | | 5.1 | 12.5 | | |
|  | 35:Psychiatric conditions | 74.7 | 69.7 | 65.1 | 73.9 | 52.0 | 54.6 | | 90.6 | 65.0 | 81.8 | 49.9 | 87.8 | 50.9 | | 73.8 | 41.1 | | 75.2 | 48.1 | | 79.5 | 65.3 | | 87.4 | 67.2 | | |
|  | 38:Diagnosis not classifiable | 17.4 | 3.5 | 6.8 | 1.7 | 6.7 | 3.9 | | <1 | 0.9 | 1.6 | 5.8 | <2 | 9.8 | | <1 | 6.1 | | 0.8 | 8.5 | | <5 | 2.1 | | 0.6 | 0.6 | | |
|  | Other | 4.3 | 15.8 | 18.1 | 13.7 | 12.4 | 16.4 | | 4.1 | 10.4 | 7.8 | 26.8 | 4.0 | 19.6 | | 6.2 | 28.4 | | 5.6 | 20.0 | | 4.9 | 12.1 | | 4.7 | 11.5 | | |
| Patient Group | |  |  |  |  |  |  | |  |  |  |  |  |  | |  |  | |  |  | |  |  | |  |  | | |
|  | Deliberate self-harm | 19.9 | 29.5 ^§^ | 36.1 | 23.7 | 45.5 | 45.2 | | 3.0 | 31.5 | 15.4 | 48.7 ^§^ | <8 | 43.7 | | 31.6 | 61.0 ^§^ | | 29.3 | 53.9 | | 4.3 | 32.3 ^§^ | | 0.0 | 28.5 | | |
|  | Other accident | 12.3 | 1.1 | 4.2 | 2.6 | 2.0 | 2.3 | | 9.8 | 7.3 | 0.7 | 5.8 | <2 | 5.0 | | 0.7 | 1.8 | | 0.7 | 1.3 | | 11.1 | 5.3 | | 15.3 | 7.6 | | |
|  | Other | 67.8 | 69.4 | 59.7 | 73.7 | 52.5 | 52.6 | | 87.2 | 61.2 | 83.9 | 45.5 | 91.0 | 51.3 | | 67.7 | 37.2 | | 70.0 | 44.8 | | 84.7 | 62.3 | | 84.7 | 63.8 | | |
| Arrival mode | |  |  |  |  |  |  | |  |  |  |  |  |  | |  |  | |  |  | |  |  | |  |  | | |
|  | Ambulance | 48.6 | 50.4 | 40.1 | 46.2 | 48.0 | 52.4 | | 35.9 | 46.7 | 60.1 | 56.4 | 56.2 | 54.3 | | 58.4 | 60.0 | | 55.3 | 59.4 | | 48.0 | 48.0 | | 48.7 | 46.6 | | |
|  | Other | 51.4 | 49.5 | 59.9 | 51.8 | 52.0 | 47.5 | | 62.9 | 52.7 | 39.9 | 42.4 | 43.8 | 41.9 | | 41.6 | 39.9 | | 44.7 | 40.6 | | 52.0 | 51.6 | | 51.3 | 52.7 | | |
|  | Unknown | 0.0 | 0.1 | 0.0 | 2.0 | 0.0 | 0.1 | | 1.2 | 0.6 | 0.0 | 1.3 | 0.0 | 3.8 | | 0.0 | 0.0 | | 0.0 | 0.0 | | 0.0 | 0.4 | | 0.0 | 0.7 | | |
| Referral source | |  |  |  |  |  |  | |  |  |  |  |  |  | |  |  | |  |  | |  |  | |  |  | | |
|  | Self-referral | 62.9 | 53.7 ^§^ | 73.9 | 56.0 | 63.2 | 45.2 ^§^ | | 75.2 | 53.1 | 59.1 | 52.5 | 55.6 | 54.1 | | 83.7 | 40.5 ^§^ | | 81.7 | 39.7 | | 69.8 | 51.4 ^§^ | | 89.2 | 54.3 | | |
|  | Emergency services | 6.1 | 19.2 | 2.4 | 14.1 | <5 | 32.8 | | 0.0 | 1.0 | 26.3 | 26.4 | 33.2 | 29.4 | | 0.0 | 34.5 | | <5 | 37.3 | | 17.8 | 12.6 | | <0.5 | 0.3 | | |
|  | Police | 4.0 | 5.5 | 6.0 | 5.5 | 8.9 | 6.6 | | 0.0 | 5.0 | 3.8 | 6.1 | 3.7 | 5.2 | | 5.6 | 6.1 | | <5 | 5.9 | | 1.4 | 5.5 | | <0.5 | 4.1 | | |
|  | Health care provider | 3.1 | 2.7 | 5.8 | 9.5 | <2 | 7.5 | | 5.2 | 32.6 | 1.4 | 2.0 | 1.1 | 2.6 | | 1.1 | 4.1 | | 0.9 | 5.8 | | 4.8 | 21.6 | | 4.9 | 33.4 | | |
|  | Other | 24.0 | 19.1 | 11.9 | 15.0 | 22.6 | 7.9 | | 19.7 | 8.3 | 9.5 | 13.0 | 6.4 | 8.7 | | 9.6 | 14.8 | | 12.4 | 11.3 | | 6.1 | 8.9 | | 5.6 | 7.9 | | |
| Discharge method | |  |  |  |  |  |  | |  |  |  |  |  |  | |  |  | |  |  | |  |  | |  |  | | |
|  | Admitted same provider | <10 | 22.6 ^§^ | 9.3 | 19.4 | 15.3 | 21.5 ^§^ | | 7.1 | 16.1 | 17.5 | 23.4 ^§^ | 13.1 | 21.9 | | 13.4 | 36.3 ^§^ | | 12.8 | 32.5 | | <10 | 19.0 ^§^ | | 8.0 | 14.7 | | |
|  | Referred other healthcare professional | <1 | 9.8 | 10.5 | 6.9 | 0.8 | 8.0 | | 12.7 | 3.9 | 0.4 | 8.3 | <0.5 | 8.3 | | 9.7 | 4.1 | | 16.3 | 5.3 | | <6 | 3.9 | | <5 | 4.0 | | |
|  | Discharged to GP | 42.7 | 25.5 | 30.2 | 21.8 | 4.3 | 20.9 | | 10.4 | 10.8 | 38.3 | 20.4 | 34.1 | 21.5 | | 54.5 | 17.5 | | 48.7 | 20.4 | | 17.8 | 16.5 | | 8.3 | 12.8 | | |
|  | Discharged no follow-up | 27.4 | 22.4 | 33.5 | 28.9 | 64.8 | 32.3 | | 58.4 | 47.8 | 17.9 | 29.6 | 19.5 | 31.0 | | 11.7 | 24.7 | | 13.3 | 25.2 | | 55.9 | 37.0 | | 71.3 | 47.5 | | |
|  | Transferred other provider | 13.6 | 6.3 | 12.1 | 9.4 | 1.9 | 4.6 | | 9.1 | 2.9 | 17.1 | 4.1 | 28.7 | 4.4 | | 0.0 | 2.9 | | 0.0 | 3.4 | | 4.6 | 5.6 | | 4.3 | 6.8 | | |
|  | Other | 6.5 | 13.4 | 4.5 | 13.7 | 12.8 | 12.7 | | 2.3 | 18.5 | 8.9 | 14.2 | <5 | 12.8 | | 10.7 | 14.4 | | 8.9 | 13.2 | | 6.5 | 18.0 | | <5 | 14.1 | | |
| Note: selected pairs of results were rounded to protect patient confidentiality where group totals were small.  **^Ϯ^** St George’s University Hospitals NHS Foundation Trust was excluded as data counts were too sparse | | | | | | | | § Group characteristic failed chi-square test of no difference between the treated trust and the synthetic controls in the pre-implementation period.. Only significant differences in the pre-period are noted  **^ξ^** The synthetic control trusts were weighted composites of the control trusts | | | | | | | | | | | | | | | | | | |  |  |
