# Supplementary material for: The impact of psychiatric decision units on mental health crisis care pathways: a synthetic control study
Source: PLOS Ment Health. 2025 May 2;2(5):e0000171. doi: 10.1371/journal.pmen.0000171 (PMC12798399; doi:10.1371/journal.pmen.0000171)
Supplement: S1 Fig — (DOCX) [file pmen.0000171.s004.docx]

**S1 Fig****: Matrix of graphs showing mental health trust outcomes for treated trusts (red line) and synthetic controls (blue line)**.

***A. Rate of mental health inpatient admissions***


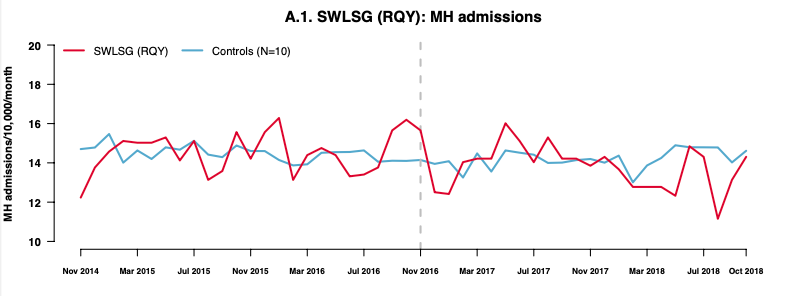

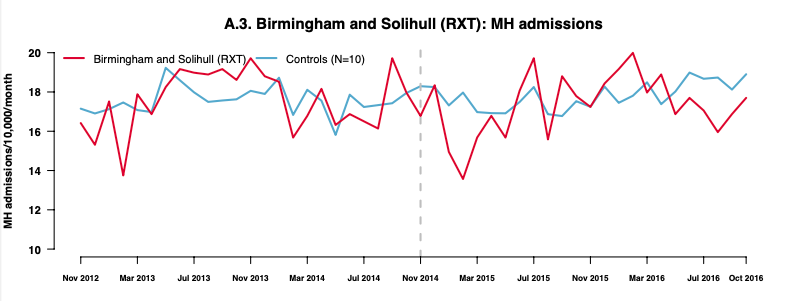


***B. Mental health inpatient admission length of stay (days)***


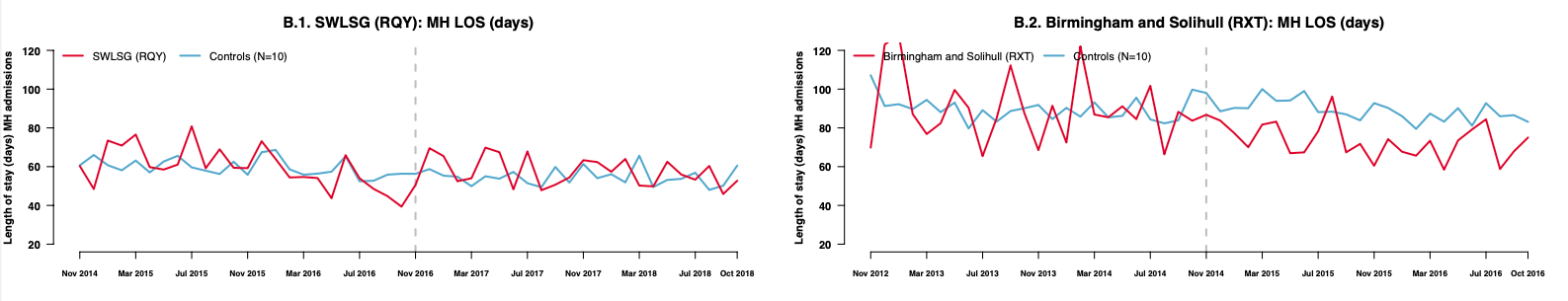


***C. Proportion of mental health admissions with a stay of less than 5 days***


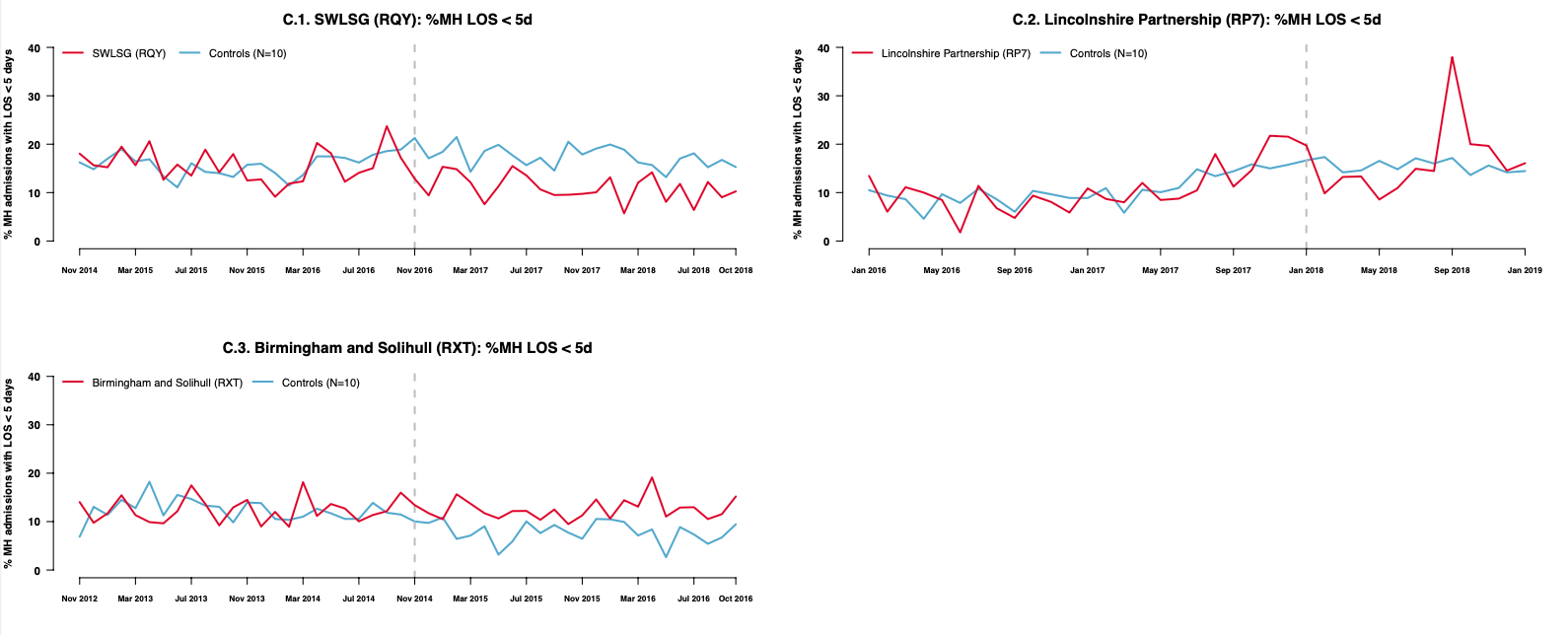


Note: The two lines are similar in the pre-intervention period (to the left of the first grey dashed line). This is by design as the synthetic control aims to track the outcome in the treated trust during this period. The difference between the two lines after PDU implementation (to the right of the grey dashed line) provides an estimate of the impact of PDU implementation on that outcome. Results are shown only for outcomes passing GSC diagnostic tests.
