# Supplementary material for: The impact of psychiatric decision units on mental health crisis care pathways: a synthetic control study
Source: PLOS Ment Health. 2025 May 2;2(5):e0000171. doi: 10.1371/journal.pmen.0000171 (PMC12798399; doi:10.1371/journal.pmen.0000171)
Supplement: S2 Fig — (DOCX) [file pmen.0000171.s005.docx]

**S2 Fig: Matrix of graphs showing acute trust outcomes for treated trusts (red lines) and synthetic controls (blue lines)**.

***A. rate of ED psychiatric attendances per 10,000 trust population per month***

***B. Proportion of ED psychiatric attendances with a stay of less than 4 hours***

***C. Proportion of ED psychiatric attendances with a stay of less than 12 hours***

***D. Proportion of ED psychiatric attendances admitted***

***E. Proportion of ED psychiatric attendances with conveyance to hospital by ambulance or police***

***F. ED psychiatric wait time (hours).***

Note: The two lines are similar in the pre-intervention period (to the left of the first grey dashed line). This is by design as the synthetic control aims to track the outcome in the treated trust during this period. The difference between the two lines after PDU implementation (to the right of the grey dashed line) provides an estimate of the impact of PDU implementation on that outcome. Results are shown only for outcomes passing GSC diagnostic tests.
